# A protocol for measuring sleep at home in children with autism using EEG headbands

**DOI:** 10.21203/rs.3.rs-7419242/v1

**Published:** 2025-09-02

**Authors:** Paige Hickey Townsend, Lindsey Jones, Anabella Tolosa, Nicole Massa, Nicole A. Simon, Dimitrios Mylonas, Ann M. Neumeyer, Dara S. Manoach

**Affiliations:** Massachusetts General Hospital; Massachusetts General Hospital; Massachusetts General Hospital; Massachusetts General Hospital; Massachusetts General Hospital; Massachusetts General Hospital; Massachusetts General Hospital; Massachusetts General Hospital

**Keywords:** Autism, sleep, EEG, wearable device, desensitization

## Abstract

**Background:**

Autism spectrum disorders (ASD) are a group of neurodevelopmental disorders characterized by social and communication challenges and the presence of restricted interests, repetitive behaviors, and atypical sensory processing. Sleep disturbances are prevalent in ASD and worsen daytime functioning, yet their neural bases remain poorly understood. We hypothesize that thalamocortical circuit dysfunction contributes to poor sleep in ASD. To test this hypothesis, we employ sleep EEG to determine whether sleep spindles, biomarkers of thalamocortical circuit function, are reduced in ASD and correlate with sleep quality. Since laboratory-based overnight sleep studies are costly, burdensome, and particularly problematic for individuals with ASD, we use wearable EEG headbands to record sleep in the home. This paper describes the methodology of our ongoing study of sleep in adolescents with ASD, including those with profound autism. An optional desensitization protocol helps participants gradually acclimate to the headband, promoting inclusion of individuals with sensory sensitivities and greater support needs.

**Methods:**

We plan to enroll 80 adolescents with ASD and 80 of their typically developing peers. Participants and their families are trained remotely to use a wearable EEG headband. They also complete assessments and questionnaires. Participants who need time to acclimate follow a personalized 2-week desensitization protocol with a practice headband. Sleep is monitored at home for 3 consecutive nights, the first of which is for adaptation to sleeping with the headband. Sleep data quality is checked daily and remote technical support is provided as needed.

**Discussion:**

At-home sleep monitoring enables larger samples that are needed to characterize the neural underpinnings of sleep disturbances across the autism spectrum. The personalized desensitization protocol promotes inclusivity by accommodating individuals often excluded from research due to sensory or behavioral challenges. These methods are adaptable for other conditions and research goals beyond ASD, such as longitudinal studies across development or characterizing sleep in other neurodevelopmental disorders. Desensitization protocols may also increase the tolerability of diagnostic lab-based sleep studies and other medical procedures, improving both patient experience and quality of care.

## Background

Autism spectrum disorder (ASD) is a neurodevelopmental disorder characterized by challenges with social interaction and communication, and the presence of restricted interests, repetitive behaviors, or atypical sensory processing (i.e., hyper/hypo-sensitivities) [1]. Sleep disturbances afflict up to 80% of children with ASD [2] and exacerbate core symptoms [3, 4], maladaptive behaviors [5, 6], and learning and attention deficits [7]. Despite the links between poor sleep and worse daytime functioning, the neural mechanisms underlying sleep disturbances in ASD remain largely unknown. This reflects, in part, the difficulty of laboratory-based polysomnography (PSG) studies, which are costly and burdensome to both participants and staff. This paper describes the methodology of an ongoing study of sleep neurophysiology in adolescents with ASD that addresses these challenges by collecting sleep data in the home using wearable electroencephalography (EEG) headbands and implementing a desensitization protocol to acclimate participants to wearing the EEG headband overnight. These methods are scalable, cost-effective, and facilitate larger, more inclusive sleep studies, including clinical trials. They hold the promise of illuminating the bases of sleep disturbances – a critical step towards the development of effective intervention.

A growing body of work demonstrates that individuals with ASD exhibit abnormal brain rhythms during sleep, most notably a reduction in sleep spindles – a defining oscillation of non-rapid eye movement (NREM) sleep. Spindles are generated in the thalamus and are propagated to the cortex via thalamocortical circuitry, where they are often synchronized with cortically generated slow oscillations (SOs). The reduction of spindle density (#/min; [8–10]) in ASD suggests a dysregulation of thalamocortical interactions [11]. Given the thalamus’ role in gating the relay of sensory information from the senses to the cortex, abnormal thalamocortical interactions could contribute to both sleep disturbances and waking manifestations of ASD. Sleep spindles are theorized to protect the sleeper from being awakened by external noise [12]. Findings that brain responses to noise are dampened during spindles [13, 14] and that people with fewer spindles are more likely to be awakened by noise [15] raise the possibility that the spindle deficit in ASD contributes to worse sleep quality. During wake, impaired sensory gating may contribute to sensory overload and sensitivities. Modulating thalamocortical interactions pharmacologically or via noninvasive brain stimulation has the potential to increase spindles and to improve sleep quality and daytime functioning.

Sleep oscillations are traditionally recorded with laboratory-based PSG, which includes scalp EEG, electrooculogram (EOG), and electromyogram (EMG) recordings. However, the laboratory setting and PSG equipment can be problematic for individuals with ASD. Children with ASD are three times more likely to have difficulty tolerating PSG [16] and take longer to acclimate to the equipment than their typically developing (TD) peers [16, 17]. Disruption to nightly routines and the unfamiliar setting may trigger anxiety and discomfort [18]. People with ASD may also be particularly sensitive to ‘first night effects’ to sleeping in a new environment, which include worse sleep quality and consequently, reduced functioning the next day. One or more adaptation nights may be required to acclimate [19]. Overnight stays in a lab interfere with routines and can be logistically impractical for many families. Sleep studies are labor intensive for researchers, and the cost of equipment, personnel, and space can be prohibitive. Research teams may also lack experience working with individuals with behavior and communication impairments, which can make it difficult to anticipate and mitigate problems that arise.

For the approximately 26.7% of people with ASD who are profoundly affected [20] (i.e., intellectual disability, minimal or no verbal ability, and/or high daily support needs), sleep studies are even more challenging [21]. Their difficulty understanding and complying with instructions, and maladaptive (e.g., aggression, self-injury, meltdowns) or sensory behaviors (e.g., mouthing or chewing), often leads to exclusion from research [22]. The proportion of studies including profound autism has decreased significantly since the 1990s across a range of disciplines [23]. Despite their significant sleep challenges [24, 25], there are few EEG sleep studies in profound autism [8]. Their exclusion limits our understanding of the heterogeneity of sleep abnormalities and contributes to small sample sizes that do not represent the entire autism spectrum.

Wearable EEG headbands used at home offer a practical alternative to lab-based PSG, potentially broadening participation and inclusion in sleep studies of ASD. Commercially available sleep EEG headbands measure sleep quality and physiology and have been validated against PSG for sleep staging [26]. They are also relatively inexpensive, user-friendly, and more comfortable than PSG. Although an adaptation night is still advisable to acquire representative sleep data, home monitoring alleviates the anxiety associated with laboratory sleep studies and interferes less with nightly routines. For participants with sensory sensitivities, a structured desensitization protocol can increase the likelihood of successful study completion [17, 27–35]. This involves gradually introducing the equipment over time in a series of incremental steps. (Desensitization training can also help participants complete overnight PSG recordings [17, 32, 34].) The use of EEG headbands with a desensitization protocol enables the inclusion of larger, more representative samples, which is necessary to understand sleep disturbances across the autism spectrum and to inform treatment development.

## Methods/Design

### Study aim and hypotheses

The aim of this study is to characterize sleep oscillations in adolescents with ASD, including profound autism. To achieve this goal, we monitor sleep in the home using a wearable EEG headband and, when necessary, employ a desensitization protocol to gradually acclimate participants to wearing it overnight. We hypothesize that dysfunctional thalamocortical circuitry and subsequent spindle deficit underlies sleep disturbances in ASD. Using sleep spindles as physiological biomarkers of circuit function, we test the predictions that this circuitry will be atypical in ASD (reflected as reduced spindle density) and correlate with worse sleep quality (i.e., more wake after sleep onset (WASO) and a higher sleep fragmentation index (SFI)).

### Study Design

**Figure 1:**
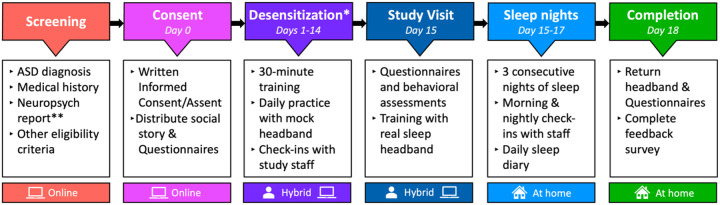
Study timeline. Interested participants/families complete online screening to confirm eligibility and provide informed consent virtually via video call. Families have the option to begin the study with a desensitization protocol. Those completing desensitization are sent a mock headband to practice with for up to 2 weeks. Families complete a 30-minute training call and respond to check-ins with the team. Once acclimated to sleeping with the headband, participants and their families complete a 1.5 hour study visit either in-person or remotely (hybrid), during which they complete standardized assessments and are trained on how to use the real EEG headband. Participants sleep wearing the headband for 3 consecutive nights. At the end of the study, the equipment is returned by mail in a pre-paid box and families complete a feedback survey. * Desensitization is optional and intended for those who need additional time to acclimate to sleeping in the headband. ** Neuropsychological reports are only obtained from ASD and profound participants.

The headbands are mailed to participants’ homes where we collect EEG recordings across three consecutive nights of sleep. Training on setting up and using the headbands is conducted either during an in-person study visit or via video call. Participants with sensory sensitivities or those needing additional adaptation time receive a practice “mock” headband (i.e., an identical headband with the electronics deactivated) and complete a modular desensitization protocol. The goal of the protocol is to help participants to sleep comfortably in the headband. The study timeline is presented in Figure 1.

### Participants

We plan to enroll 80 12–19 year old adolescents with ASD and 80 TD peers. Given an anticipated 10% attrition rate, we expect to acquire complete datasets from 72 per group. Participants are recruited from the Lurie Center for Autism in Lexington, MA, the Simons Foundation Powering Autism Research (SPARK) initiative via Research Match, and Mass General Brigham’s online research database platform, Rally. Participants (with the exception of profound autism) and parents/guardians of minors under 18 must be able to converse and respond to questionnaires in English.

#### ASD.

Clinical diagnoses of ASD are confirmed through chart review, parental responses to the Social Communication Questionnaire (SCQ) [36], and the completion of the DSM-V checklist. All ASD participants must meet criteria for ASD on the DSM-V based on chart review or have an SCQ score ^3^15. Participants with known genetic syndromes associated with ASD, diagnosed sleep disorders (except insomnia), uncontrolled seizures (one or more within the last week), other neurological conditions, or uncontrolled chronic medical conditions are excluded.

#### Profound autism.

In addition to meeting criteria for ASD, participants with profound autism must also meet at least one of the following criteria: minimally verbal (<1^st^ percentile on the Expressive Vocabulary Test (EVT-3) [37]; or uses no words, only single words, or short phrases (2–3 words) on a daily basis per parental report), intellectual disability (nonverbal intelligence quotient; NVIQ < 70), significant maladaptive behaviors (total score on the Aberrant Behavior Checklist (ABC [38] > 49), or sensory sensitivities.

#### Typically Developing (TD).

TD participants must not have a diagnosed neurodevelopmental disorder (e.g., ADHD), a first-degree biological relative with ASD, an SCQ score ^3^15, or a diagnosed psychiatric (e.g., currently medicated for depression or anxiety), sleep, or neurological disorder (including seizures), or an uncontrolled chronic medical condition expected to affect sleep.

#### Ethics approval and consent to participate.

Informed consent and assent are provided at study onset by the participant (^3^18 years old) or a parent/guardian (<18 years). Written assent is also required for participants under 18. In the profound autism group, a parent or legal guardian provides informed consent regardless of age and written assent is obtained from participants when possible. The study is funded by the Simons Foundation Autism Research Initiative, the Autism Science Foundation, and CureShank. The study protocol was approved by the Mass General Brigham Institutional Review Board. The research is conducted in accordance with the Declaration of Helsinki.

### Headband selection

We considered a number of commercially available EEG headbands, several of which have shown good agreement with PSG for sleep staging [26]. Our evaluation criteria included price, electrode montage, availability of raw data, accuracy of characterizing sleep architecture and sleep physiology, comfort, and ease of setup and use. We also considered durability and the presence of detachable components given the possibility of participants mouthing, throwing, or banging the headband. We validated four headbands against PSG for sleep staging and compared spindle and SO detection. We chose the Dreem 3 EEG headband (DREEM, Inc., acquired by Beacon Biosignals Inc. in 2023). Dreem 3 has three frontal (FP1, F7, F8) and two occipital electrodes (O1, O2), along with an accelerometer. Data are stored locally on the headband and automatically uploaded to the company’s server when the headband is charged. Dreem 3 includes proprietary algorithms for assessing signal quality, sleep staging, and estimating body position and the proportion of time the headband was not worn. Researchers can access data through the Dreem 3 web-based platform. Although Dreem 3 was selected for our study, the landscape of commercially available EEG headbands is continually evolving, with new devices being released and prices and designs of existing devices changing. Thus, researchers should consider which headband best fits the needs of their study.

### Screening

Interested families complete a screening call with the study team to determine eligibility. Parents (or participants >18 years old in the non-profound group) are asked about their child’s medical history, including any diagnoses (e.g., ASD, genetic syndromes, anxiety, depression, epilepsy), medications, and sleep. Parents complete the SCQ [36] to confirm their child’s ASD diagnosis or lack thereof (for TD) and either the Pediatric Sleep Questionnaire (PSQ; ages 12–17 [39]) or the STOP-BANG [40] (ages 18+) to exclude suspected sleep apnea. For independent adults (ages 18+), the Autism Quotient (AQ; [41]) can also be used to confirm ASD diagnosis.

Gathering additional information about participants from their families prior to participation can be the difference between successful completion and withdrawal [29, 33, 35, 42, 43]. If desensitization is needed, parents complete a more in-depth screening interview about their child’s expressive language skills, maladaptive behaviors that may occur during the visits (e.g., elopement, self-injury) or while practicing with the headband, sensory sensitivities (e.g., difficulty tolerating things on their head) or sensory seeking behavior (e.g., mouthing), and motivators that might help their child succeed (**Supplement 1**) [33, 35, 43]. This information helps study staff personalize the desensitization training and prepare for in-person visits. For example, familiar strategies, such as timers, visual schedules, and sticker charts, can be leveraged to encourage practice with the mock headband, and preferred rewards could be offered during breaks, after completing each desensitization module, and after completing the study. In depth screening can also help researchers predict obstacles or challenges that individuals might encounter during the study and prepare mitigation strategies (e.g., providing a reward for not mouthing the device or an alternate object that can be mouthed instead [33, 35, 43].

### Desensitization

**Figure 2:**
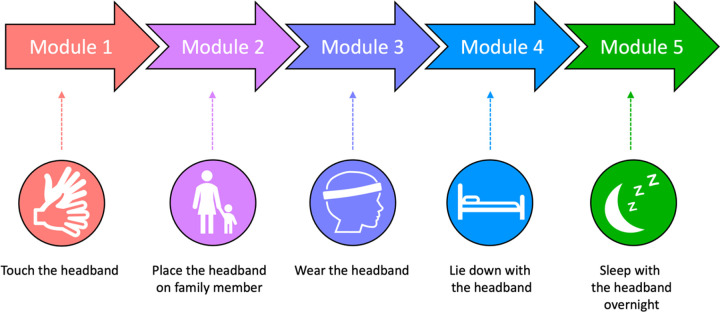
Desensitization Modules.

Participants may complete an optional two-week desensitization protocol to acclimate to wearing the headband. Families practice five modules at home with the mock headband: (1) touching the headband, (2) having a family member model wearing the headband, (3) having the child wear the headband for at least 5 minutes, (4) having the child lay down wearing the headband for at least 5 minutes, and (5) sleeping in the headband overnight (Figure 2).

At the beginning of desensitization, families attend a 30-minute meeting (either in-person or virtual) with the study team and a behavioral specialist. They receive a social story outlining the five modules (**Supplement 2**) and are introduced to the headband. The study team observes the participant’s initial response to the headband and has them attempt the first 4 modules to determine which one they should begin with. Families are given instructions and tips-and-tricks for practicing at home (**Supplement 3A**). Families are encouraged to practice at least 3 times per day and are asked to log their child’s progress, including which module they practiced, how long they practiced, and their child’s level of comfort and cooperation (**Supplement 3B**). To avoid developing a negative association to the headband, families are asked to stop practicing if their child becomes uncomfortable or resistant and allow the study team to help troubleshoot. Since cooperation and comfort may look different for each child, families complete a worksheet identifying behaviors they observe when their child is uncomfortable/resistant in other situations to inform their rating of comfort/cooperation (**Supplement 3C**). Once the child successfully completes a module three times in a row (Modules 1–4), they advance to the next module. Desensitization is complete once the child sleeps in the headband overnight one time (Module 5).

The study team monitors progress through daily text check-ins (**Supplement 3D**) and a phone check-in after the first week. Based on the child’s initial tolerance of the headband and the family’s available practice time, desensitization may extend up to a month. Children with greater sensory sensitivities may need more time adapt to the equipment [29, 32–35, 42, 43]. Some may also benefit from breaking down the desensitization modules into smaller intermediate steps. Providing opportunities for breaks and moving at the pace of the child will further improve quality data [33]. While a flexible desensitization period helps support participants, parents have demanding schedules and limited time to dedicate to the study. The study team balances regular engagement with families to provide support and encourage continued participation with efforts not to overburden families with too frequent or time-consuming check-ins. Check-ins are adapted to their schedule, preferred method, and frequency.

### Study Visit

**Figure 3:**
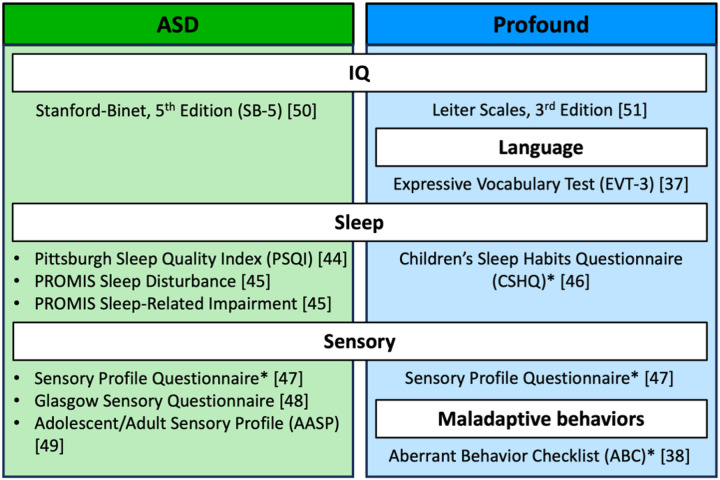
Questionnaires and assessments. *Completed by participant’s parent.

Prior to the study visit, families are emailed an individualized social story to help them prepare for the visit and upcoming sleep recordings (**Supplement 2**) and remote participants are mailed the headband. During the visit participants are trained to use the EEG headband. The study team demonstrates how to download the phone app, place the headband, check the signal quality, start/stop the recording, and recharge the headband. The study team inspects the fit of the headband (i.e., ensuring it is placed correctly and fits snugly on the head, providing extenders if it is too small) and explains how to obtain good quality data (i.e., removing hair from underneath the headband, wearing a cloth headband over the EEG headband to keep it in place). Printed instructions for setting up the headband and nightly/morning checklists are provided to families for reference (**Supplement 4A**). Participants and their families also complete questionnaires and neuropsychological assessments to assess their sleep [44–46], sensory sensitivities [47–49], language skills [37], and maladaptive behaviors [38] (Figure 3). In-person participants are administered an assessment of verbal and non-verbal IQ [50] or the Leiter International Performance Scale, 3^rd^ edition [51]. Remote participants’ non-verbal IQ estimates are obtained from a previous neuropsychological report or a three-year evaluation for the child’s individual education plan.

### Overnight EEG Acquisition and Sleep Diary

Participants sleep with the headband for three consecutive nights at home, with the first being an adaptation night. We aim for weeknights to have a more consistent sleep schedule. Each evening, study staff check-in with the participant and/or their family by video call, phone call, or text message to offer technical support for headband set-up and placement. Each morning, participants and/or their families stop the recording, charge the headband, and complete a sleep diary and questionnaire about headband comfort and usability (**Supplement 4B**). When possible, profound autism participants complete questionnaires about the comfort of the headband that have been adapted by using pictures and icons (**Supplement 4C**). Sleep diaries are completed by their parents.

### Data Quality Evaluation

**Figure 4:**
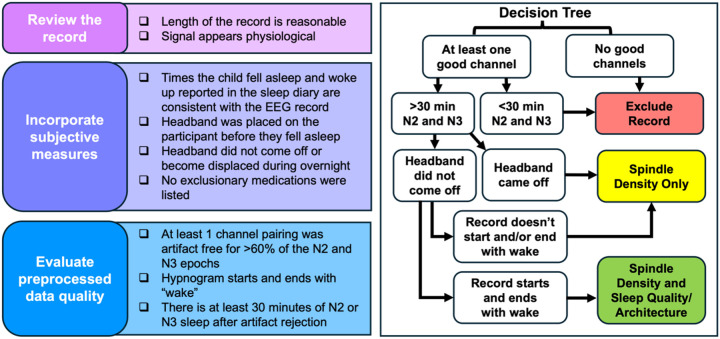
EEG quality checklist. EEG data quality is evaluated by the study team each morning. Researchers visually review EEG data, consider responses to the sleep diary and questionnaires, and run initial preprocessing on the data to ensure it is usable. If the headband remained on the head all night, EEG records with good channel quality are used for estimating sleep quality and sleep architecture. Spindle density and sleep physiology during NREM sleep is evaluated if there is at least one continuous bout of stage 2 (N2) and stage 3 (N3) sleep. If data quality is poor, the research team contacts the family to troubleshoot. If there are no usable records after 3 nights, participants are given the option to sleep wearing the headband for a fourth night.

Each morning the study team downloads raw EEG and accelerometer data from the Dreem 3 platform to assess data quality by checking that: 1) the raw EEG and accelerometer data are an appropriate length; 2) signal looks physiological rather than artifactual; 3) sleep diary responses are consistent with the EEG recording; 4) and the headband remained on throughout the night. (If the headband comes off, sleep quality and architecture are not calculated). The EEG data are sleep staged using USLEEP [52] and then preprocessed using Luna (http://zzz.bwh.harvard.edu/luna/) to identify epochs containing artifacts. Channels with >40% epochs identified as artifactual are flagged as being poor quality and removed. We also inspect the whole-night time-frequency spectrograms aligned with the participant’s hypnogram, and the power spectra of NREM stage 2 (N2) and NREM stage 3 (N3) sleep before and after preprocessing. Usable records have at least one channel with good data quality, and at least 30 minutes of artifact-free N2 and N3 sleep. See Figure 4 for the data quality checklist. If the participant reports that the headband was uncomfortable or if the data were poor quality, the study team contacts the family to troubleshoot. Medications that are taken are documented in the sleep diary each night.

### Study Completion

At the end of the study, participants return all equipment by mail in a prepaid box provided by study staff along with a return label, packing tape, and packing list. In addition to monetary compensation for their participation, participants receive a personalized completion certificate that includes a sleep spindle recorded during the study (**Supplement 5**) and a copy of “*Why Sleep*”, an article about the functions of sleep written for children [53]. Families also have an opportunity to provide feedback about their study experience.

## Discussion

Large-scale sleep studies in ASD present unique challenges but are crucial for developing and evaluating interventions for poor quality sleep. Here we describe an approach to studying sleep oscillations in individuals with ASD using a commercially available EEG headband worn overnight at home. The primary aim of the study is to characterize sleep quality, architecture, and physiology in a large sample of adolescents with autism, including those with profound autism. As the sleep oscillations that we measure map onto specific neural circuits, this study will elucidate the neural circuitry underlying abnormal sleep physiology in ASD and test the hypothesis that it underlies poor sleep quality. This protocol enables larger, more inclusive samples in research and clinical trials aimed at developing and evaluating mechanistically targeted treatments for sleep disturbances in ASD.

Due to the heterogeneity of ASD, a one-size-fits-all approach to sleep studies may limit who is able to participate and restrict sample sizes. To promote inclusion and achieve larger samples, several strategies can be implemented to accommodate individuals with greater support needs. For example, including a behavioral specialist with expertise in ASD during study design and execution can facilitate data collection and improve the participant’s experience in the study. They can recommend modifications and tailor procedures to each participant’s needs [29, 35]. They can also provide behavioral training to prepare study staff for successful interactions with participants. Considering intellectual and communication abilities [35, 43] is also necessary to determine which questionnaires and assessments are appropriate. If subjective report measures are not feasible, parental report or questionnaires with pictures may be alternatives. Neuropsychological assessments that do not rely on receptive or expressive language, such as the Leiter [51], may be better suited to characterize cognitive function. Social stories, visual schedules, and videos can clarify study procedures, reduce uncertainty, and ease transitions between tasks [29, 33, 34, 54]. Study instructions should be concise, use simple language, and incorporate visuals or modeling. Providing materials in different formats – such as video and printed social stories – allows families to choose the most effective approach for their child. Consulting with families during protocol development [29] and personalizing the research experience as much as possible [42] will promote successful participation.

This protocol also enhances accessibility and inclusivity by allowing overnight EEG data to be collected remotely in the home. This removes geographical constraints that limit participation for families living in rural areas or far from the laboratory. Remote overnight EEG data collection enables studies of rare populations. For example, we are also using this protocol to study sleep in individuals with monogenic syndromes associated with ASD, specifically Phelan-McDermid, Fragile X, and 16p11.2 deletion syndrome. These rare genetic syndromes account for <0.02% of the population and families are geographically dispersed. Although sleep disturbances are highly prevalent in these syndromes [55–58], their sleep physiology has seldom, if ever been studied. Collecting sleep data in the home has allowed us to expand our recruitment to across the US. Our desensitization protocol has also facilitated greater participation.

The methodology described here can be adapted for a wide range of other conditions and research goals beyond ASD. For example, this approach is well suited for longitudinal studies that track sleep across typical and atypical development or characterizing sleep in other neurodevelopmental disorders. Although wearable EEG devices allow researchers to study sleep in disorders like apnea, narcolepsy, and epilepsy remotely, diagnoses still require hospital or lab-based PSG. In these cases, our desensitization protocol, social stories and other practice materials, could be adapted to help patients acclimate to the sensory components of the PSG equipment and the hospital environment prior to their study, increasing the likelihood of successful PSG acquisition. Similar protocols could also be developed for increasing tolerability of other medical procedures such as physical exams, blood draws, vaccinations, and imaging (e.g., EEG, MRI, CT). Integrating desensitization methods into clinical practice could improve both patient experience and quality of care.

We are continuing to refine our study protocols based on family feedback about desensitization training, headband comfort and usability, and overall study experience. Understanding hurdles helps us hone our current strategies for overcoming them and add new tips-and-tricks for participants. We plan to streamline surveys and materials into a smartphone app, providing families with a centralized and easily accessible platform for practice logs, sleep diaries, checklists, surveys, and reminders. This will reduce burden on families and researchers and facilitate individualization to encourage participation.

## Supplementary Material

Supplementary Files

This is a list of supplementary files associated with this preprint. Click to download.

- EEGHeadbandProtocolMSSupplement.docx

## Data Availability

Data sharing is not applicable as no datasets were generated or analyzed for this article.

## Abbreviations

AASP: Adolescent/Adult Sensory Profile
ABC: Aberrant Behavior Checklist
AQ: Autism Quotient
ASD: autism spectrum disorder
CSHQ: Children’s Sleep Habits Questionnaire
EMG: electromyogram
EOG: electrooculogram
EVT-3: Expressive Vocabulary Test, 3rd Edition
N2: non-rapid eye movement stage 2 sleep
N3: non-rapid eye movement stage 3 sleep
NREM: non-rapid eye movement
NVIQ: nonverbal intelligence quotient
PSG: polysomnography
PSQ: Pediatric Sleep Questionnaire
PSQI: Pittsburgh Sleep Quality Index
SB-5: Stanford-Binet, 5th Edition
SFI: sleep fragmentation index
SCQ: Social Communication Questionnaire
SPARK: Simons Foundation Powering Autism Research
SO: slow oscillation
STOP-BANG: Snoring, Tiredness, Observed apnea, Pressure (high blood pressure), BMI (Body Mass Index) over 35, Age over 50, Neck circumference over 40 cm, and Gender (male)
TD: typically developing
WASO: wake after sleep onset
